# A Web-Based Lifestyle Medicine Curriculum: Facilitating Education About Lifestyle Medicine, Behavioral Change, and Health Care Outcomes

**DOI:** 10.2196/mededu.7587

**Published:** 2017-09-11

**Authors:** Elizabeth Pegg Frates, Ryan C Xiao, Deepa Sannidhi, Yasamina McBride, Tracie McCargo, Theodore A Stern

**Affiliations:** ^1^ Department of Physical Medicine and Rehabilitation Spaulding Rehabilitation Hospital Charlestown, MA United States; ^2^ Harvard Medical School Harvard University Boston, MA United States; ^3^ Division of Family Medicine Department of Family and Preventative Medicine University of California San Diego La Jolla, CA United States; ^4^ Harvard Extension School Harvard University Cambridge, MA United States; ^5^ Massachusetts General Hospital Boston, MA United States

**Keywords:** life style, health behavior, healthy lifestyle, health education, health promotion, wellness programs, curriculum, self efficacy, young adult, education, medical, undergraduate, health knowledge, attitudes, practice, universities, mental health, adolescent, risk reduction behavior, humans

## Abstract

**Background:**

Lifestyle medicine is the science and application of healthy lifestyles as interventions for the prevention and treatment of disease, and has gained significant momentum as a specialty in recent years. College is a critical time for maintenance and acquisition of healthy habits. Longer-term, more intensive web-based and in-person lifestyle medicine interventions can have a positive effect. Students who are exposed to components of lifestyle medicine in their education have improvements in their health behaviors. A semester-long undergraduate course focused on lifestyle medicine can be a useful intervention to help adopt and sustain healthy habits.

**Objective:**

To describe a novel, evidence based curriculum for a course teaching the concepts of Lifestyle Medicine based on a web-based course offered at the Harvard Extension School.

**Methods:**

The course was delivered in a web-based format. The Lifestyle Medicine course used evidence based principles to guide students toward a “coach approach” to behavior change, increasing their self-efficacy regarding various lifestyle-related preventive behaviors. Students are made to understand the cultural trends and national guidelines that have shaped lifestyle medicine recommendations relating to behaviors. They are encouraged to engage in behavior change. Course topics include physical activity, nutrition, addiction, sleep, stress, and lifestyle coaching and counseling. The course addressed all of the American College of Preventive Medicine/American College of Lifestyle Medicine competencies save for the competency of office systems and technologies to support lifestyle medicine counseling.

**Results:**

The course was well-received, earning a ranking of 4.9/5 at the school.

**Conclusions:**

A novel, semester-long course on Lifestyle Medicine at the Harvard Extension School is described. Student evaluations suggest the course was well-received. Further research is needed to evaluate whether such a course empowers students to adopt behavior changes.

## Introduction

### College Student Health and Lifestyle Medicine Interventions

College students are an important target audience for lifestyle medicine—a medical specialty focused on evidence-based interventions for improving healthy lifestyle behaviors encompassing diet, exercise, and wellness. College health is a major concern, especially around stress management, sleep deprivation, weight management, nutrition, physical activity, and alcohol use [1-4]. College is a critical time for experimenting, exerting autonomy, and determining how to make choices that result in happiness and productivity. College also marks a critical time for the acquisition and maintenance of healthy habits.

Researchers and educators have been investigating the lifestyle choices of college students for decades, and studies demonstrate that unhealthy habits such as physical inactivity and poor nutritional choices become exaggerated in college. In one study, researchers tracked individual college students’ body weight and body composition and demonstrated an increased prevalence of student obesity from 17.5% (23/131) to 30.5% (40/131) between entering and graduating from college [5]. Given the obesity and diabetes epidemics in the United States and worldwide, teaching college students about healthy lifestyles can play an important role in preventing cardiovascular disease, cancer, and respiratory disease. In a different study examining the efficacy of a single 1-hour motivational intervention for college students on obesity, researchers found that students who participated in the intervention did not have significant body mass index (BMI) reductions at the 3-month follow-up when compared with control students [6]. They concluded that prolonged interventions may be necessary for a significant impact on student health. Addressing and undoing the unhealthy habits that prevail in college will take more than a 1-hour intervention.

Research on the health of college students has been completed by investigators around the world, including countries such as China [7,8], Ethiopia [9], Lebanon [10], Saudi Arabia [11], Qatar [12], Sudan [13], Serbia [14], Bahrain [15], Arab countries [16], Puerto Rico [17], Spain [18], and the United States [4,19,20]. College students’ health and behaviors are of interest because of the mental stress and anxiety that many students experience [21,22], as well as the use of excessive alcohol [23-25], smoking habits [26], drug use [27], weight [28-30], sleep problems [31,32], lack of physical activity [4], poor diets [33-35], and overall unhealthy lifestyles of college students [36-38].

Many interventions to increase the health and well-being of college students have been investigated. A systematic review and recent meta-analysis of computer-delivered Web-based interventions to improve depression, anxiety, and psychological well-being of college students demonstrated that cognitive, behavioral, and mindfulness interventions were effective in reducing stress [39]. In a systematic review of dietary interventions in college students, the authors’ analysis of 14 papers found that in-person interventions showed promise in improving students’ dietary behaviors, but these changes were minimal [40]. Interventions that included self-monitoring and goal setting maximized outcomes. In this same review, Web-based interventions were less effective overall but seemed to show efficacy with students who resisted change or considered changing their eating habits. This review also looked at environmental approaches that could increase the visual cues to action for selecting healthy options. There is evidence that in-person and Web-based interventions can help college students address stress and can nudge students in the direction of making healthy dietary choices.

### Impact of Lifestyle Related Coursework on College Health

Research has also focused on the effect that academic major selection and academic class selection have on the lifestyle behaviors of students. In one study, researchers examined the correlation between a college student’s chosen major and his or her dietary choices. It revealed that female nutrition majors had healthier habits and made superior food choices compared with female non-nutrition majors [41]. It is not clear whether the students who chose nutrition as a major had healthier habits before going into college, whether their education helped to inform them about healthy eating, or whether it was a combination of factors that led female nutrition students to have healthier eating habits. An examination of self-reports of students in a health sciences university in Bahrain revealed that a high percentage of health science students had unhealthy dietary habits and sedentary behaviors, with 48% (43/90) of male students physically inactive and 84% (463/552) of female students physically inactive on a daily basis [42]. This study revealed that students who focused on health sciences and on taking classes in health were not necessarily following a healthy lifestyle. Health sciences is a broad topic, and there is a vast array of courses that could be included in the curricula. Research demonstrates that a student’s interest in the topic of health does not mean that he or she adheres to a healthy lifestyle. The integration of nutrition education within the second year of medical school in the cardiovascular module was associated with improved heart-healthy eating habits [43].

A semester-long college course in lifestyle medicine could address many of the unhealthy habits that college students tend to adopt and sustain during college. Lifestyle medicine is the science and application of healthy lifestyles as interventions for the prevention and treatment of diseases such as heart disease, diabetes, stroke, obesity, dementia, and cancer, all of which are affected by lifestyle choices. It is the evidence-based specialty bridging the research and science of physical activity, nutrition, stress management and resilience, smoking cessation, sleep hygiene, social support, and other healthy habits to individuals through clinical practice in health care [44]. Lifestyle interventions include exercise prescriptions, nutrition prescriptions, stress management and resilience training, smoking cessation programs, sleep evaluations and recommendations, identifying and encouraging social connections, harnessing individuals’ strengths, and using positive emotions such as gratitude and laughter as medicine to empower individuals to reach their optimal state of health and well-being [45]. By providing a greater depth of knowledge concerning basic health information, guidelines, and research findings as well as prolonged exposure to lifestyle medicine tenets and by creating opportunities to practice healthy lifestyles throughout the semester, a lifestyle medicine class for undergraduates can empower students with the knowledge, skills, tools, and experiences necessary to adopt and sustain healthy habits for a lifetime.

With the American College of Lifestyle Medicine and the American College of Preventive Medicine working to create curricula for medical students, students in the health professions, and practicing physicians coupled with the newly formed American Board of Lifestyle Medicine creating a national board examination for certification in lifestyle medicine, lifestyle medicine is becoming mainstream for the treatment and prevention of chronic diseases such as diabetes, heart disease, obesity, stroke, and cancer. This paper aims to outline a curriculum for undergraduate- and graduate-level coursework that strives not only to impart an understanding of core lifestyle medicine concepts and skills as described in the existing literature but also to provide a place where students can reflect upon their own daily choices and work on their own behavior change project for a semester.

## Methods

### Course Goals

The overarching goal of the Introduction to Lifestyle Medicine Course was to demonstrate how lifestyle medicine principles and interventions apply to individual health behaviors, examining both short- and long-term health outcomes. Educational interventions on nutrition and exercise have been shown to improve student self-efficacy relating to various lifestyle-related preventive behaviors [46]. The Introduction to Lifestyle Medicine Course uses evidence-based principles that aim to increase student confidence, which enables students to co-create goals for themselves while progressing toward the adoption of healthy habits and completing their required behavior change project. By utilizing the coach approach, students are able to collaborate, negotiate, and partner with individuals to identify obstacles to change, strategies around the obstacles, character strengths, motivators, social supports, and methods of accountability that will enable lasting change. There is an emphasis on the student as both learner and teacher. All members of the course are acknowledged for their own life experience, and students share their wisdom during Web-based class discussions and discussion posts.

Introduction to Lifestyle Medicine Course students gain a solid understanding of the main components of lifestyle medicine. The history of the development of lifestyle medicine is the first focus of the course and provides the groundwork on which the rest of the material is built. Understanding cultural trends and national guidelines in lifestyle behaviors, such as exercise, diet, and stress management, encourages the students to consider the social ecological model of change, with a focus on how one’s environment helps to shape one’s behavior. The course brings the science from the medical literature and practical strategies from clinical experience to the students aspiring to instill healthful lifestyle behaviors in themselves, their family and friends, as well as future patients and clients. By the end of the 13-week course, the students have a solid understanding of the importance of practicing healthy habits.

### Course Topics and Rationale for Inclusion

#### Physical Activity, Nutrition, and Addiction

The topics of exercise, nutrition, and addictions come from the data revealing that Americans do not reach the recommended levels of exercise [4], do not eat the recommended quantities of fruits and vegetables [47], and suffer from addictions to nicotine, alcohol, and drugs. In the *New England Journal of Medicine* paper by Mokdad and colleagues (entitled Actual Causes of Death in the United States), the bar graph included reveals that tobacco use, physical inactivity, poor diet, and drug use comprise the top actual causes of death [48]. In addition, research has demonstrated that the majority of cancers can be prevented by lifestyle-related factors, which include refraining from smoking and alcohol use, having a healthy BMI, and engaging in adequate physical activity [49]. These are the most common lifestyle behaviors researched in the medical literature, and they are all significant contributors to morbidity and mortality rates.

#### Sleep

The connection of sleep to memory and learning, the connection of poor sleep secondary to obstructive sleep apnea to stroke, the connection of inadequate sleep to obesity, and the statistics on sleep deprivation and motor vehicle accidents (from driving while drowsy) have led to the inclusion of sleep as a major topic in the curriculum [50,51].

#### Stress

Health care expenditures are consistently higher among individuals with psychological stress [52], and stress is associated with depression, cardiovascular disease, and human immunodeficiency virus and acquired immunodeficiency syndrome [53]. Tackling the topic of stress is essential to a college course on lifestyle medicine. Positivity, gratitude, and laughter are ways to manage stress and increase creativity. Therefore, these topics are included to shine a light on them and also to provide some enjoyment for the students as they learn about laughter yoga, flow, and resiliency. A strengths-based approach to life is a relatively new concept [54] but is one that is proving to be empowering. Stress management techniques, their evidence base, and their practical implementation are emphasized in the course.

### Lifestyle Coaching and Counseling

Learning how to counsel people about lifestyle modification is the primary mode of delivery for lifestyle medicine. Motivational interviewing, appreciative inquiry, and other health coaching techniques are useful counseling methods in the clinical environment. Their use has been described in the medical literature as tools for patients to guide them to create and sustain behavioral change [55-57]. These concepts illustrate to college students that behavior change is not simply about “willpower” and “deciding to do it”—beliefs that all too often result in stigma toward self and others who need to change their behavior [58].

### Alignment With Core Competencies

For lifestyle medicine, the core competencies for primary care physicians were recommendations by a blue ribbon panel, which comprised physicians from the American Medical Association, the American Osteopathic Association, the American Academy of Family Physicians, the American College of Physicians, the American Academy of Pediatrics, the American College of Preventive Medicine, and the American College of Lifestyle Medicine. They are outlined in Textbox 1 and were originally delineated in the *Journal of the American Medical Association* [59]. The Introduction to Lifestyle Medicine Course addresses all of the 15 core competencies except for application of office systems and technologies to support lifestyle medicine in the office setting. This competency was not relevant for students who were not currently practicing medicine and was not included in the course curriculum. A practicing lifestyle medicine physician should possess the following: knowledge, skills, attributes, and values.

The Web-based weekly class sections invite the students to apply the material discussed in class to themselves and their own lives (See Table 1). These periods of reflection allow the students to consider how they might make healthy changes while in college.

This Web-based, distance learning college lifestyle medicine course also includes a behavior change project because knowledge is powerful, but it is not powerful enough to instill lasting change. Thus, as part of the required work, the students examine and work on their own habits. Students are encouraged to focus on a behavior or healthy habit that they are seeking to adopt for themselves. They are asked to log their current activity and situation with that particular behavior, noting triggers, rewards, and emotions around it. Then, they are asked to create a behavior change plan with SMART (specific, measurable, achievable, results-focused and time-bound) goals and to establish a tracking and accountability methodology that works for them. At the end of the semester, the students write a brief (3-5 pages) report on their experience of trying to change a behavior. Review of these reports reveals that some students in the class quit smoking, lost weight, added exercise into their routines, sat for shorter periods of time, increased their ability to handle stress, added fruits and vegetables to their diets, and cut down on their alcohol intake.

## Results

The Harvard Extension School has been offering a course in lifestyle medicine since 2014. The course was offered as a hybrid course in 2014 and 2015, meaning that students could either take the entire course from a distance or they could choose to attend lectures live. In 2016, the course was completely Web-based. In total, 75 students from across the United States and around the globe took the course during the first year; 2 years later, in 2016, there were 111 students enrolled in the course, which marked it as one of the most popular classes at the school that semester. The majority of the students were undergraduates, with the second largest cohort being masters graduate students, mostly in psychology. Practicing physicians, retired lawyers, engineers, investment bankers, rabbis, musicians, therapists, and nurses have completed the course. At its inception, the course was offered as a psychology course with psychology credits, but in the year 2016, it was also approved for biology credit, which encouraged more premedical students to enroll in the course. The course was well received, gaining one of the highest rankings at the school with a 4.9 out of 5 (5 being the highest possible rating) in all 3 years.

## Discussion

There is a health care crisis in the United States and worldwide, with epidemics of obesity and diabetes. The new medical field of lifestyle medicine has the potential to help millions of adults to reverse common chronic conditions such as heart disease, high blood pressure, diabetes, obesity, back pain, and inflammatory arthritis and to help millions of children and adolescents to prevent the development of these chronic conditions. With a board certification process in place for 2017 and physicians lining up for training in this field, lifestyle medicine is sure to play an important role in the future of health care.

Of course, the pursuit of healthy lifestyles involves both individual choices and social/institutional pressures, which include, for example, easier access to vending machine snacks rather than produce and social support (or lack thereof) [2]. Although our curriculum focuses on individual progress and change, the knowledge gained may help empower students, patients, and consumers to push for institutional changes that can facilitate healthier lifestyle choices. As such, the education system can play an important role in solving our medical and health care crises. Teaching college students about healthy lifestyles can help inform them about the rationale behind national guidelines. Although readings and lectures can teach students about lifestyle medicine, the opportunity to practice healthful behaviors and to work on changing unhealthy behaviors truly allows students to appreciate the process, challenge, and rewards of change.

The Harvard Extension School’s Web-based, distance learning course, Introduction to Lifestyle Medicine, provides a novel, evidence-based curriculum that teaches basic lifestyle medicine knowledge, skills, and tools to undergraduates at an established university that is available worldwide. A variety of lifestyle medicine guidelines are conveyed to undergraduates in a Web-based class format and have been received favorably, which is evident by the official student evaluations and their rankings submitted to the school administrators. Textbox 1 demonstrates the core competencies for lifestyle medicine. As seen in Table 1, the college curriculum meets many of the core competencies for prescribing lifestyle medicine.

Over the past 3 years, the Lifestyle Medicine course has been completed during a semester block. The strength of the course lies in the breadth of its curriculum. Central to the success of the curriculum is the behavior change strategy using the coach approach, defined as communicating with the patient in a nonjudgmental way that evokes the wisdom within patients, respects their autonomy, builds their self-efficacy, honors them as the expert in their own lives, and provides realistic strategies to help them adopt and sustain healthy habits for a lifetime. Students in the course come away with tools for behavior change that can be applied within the context of their daily lives.

The medical literature demonstrates that providers who practice behavior change are more likely to be successful when delivering the advice to their patients [60,61]. Thus, if students who complete the course go on to become certified lifestyle medicine practitioners, they will have already experienced what it takes to change a behavior and will be able to relate to patients. If the students do not plan to be lifestyle medicine practitioners, they will have the tools to help loved ones who desire to make a change in their daily habits. Moreover, the students themselves will fully understand the change process and the power of healthy living. Providing this course to students of all ages, located all over the globe, fosters connections across generations and across oceans and continents. A research study examining the health behaviors of students completing this Lifestyle Medicine course will help to determine whether the course will empower students to adopt behavior changes years after the course is over.

**Lifestyle Medicine Competencies for primary care professionals.**

1. Leadership (2 competencies)

- Promote healthy lifestyle behaviors (emphasized in week 1 and addressed in all other weeks).
- Practice healthy lifestyle behaviors (emphasized in week 1 and in the behavior change project due at the end of class).

2. Knowledge (2 competencies)

- Demonstrate knowledge that lifestyle can positively affect health outcomes (emphasized in all weeks, stressing specific behaviors during correlating weeks, such as exercise in week 4).
- Describe ways in which physicians can effect health behavior change (emphasized in all weeks, stressing specific behaviors during correlating weeks, such as nutrition in week 5).

3. Assessment skills (3 competencies)

- Assess social, psychological, and biologic predispositions (emphasized in all weeks).
- Assess readiness to change (emphasized in weeks 2 and 3 with behavior change, goals, and accountability and then addressed in each week thereafter. Case studies reinforce these principles).
- Perform lifestyle medicine focused history of present illness, physical exam and relevant anthropometric and laboratory testing (emphasized in week 1 and in each week thereafter).

4. Management skills (4 competencies)

- Use nationally recognized practice guidelines (completed for each week, such as exercise guidelines in week 4 and nutrition guidelines in week 5).
- Establish effective relationships with patients (emphasized in weeks 2 and 3 with behavior change, goals, and accountability and addressed in each week thereafter. Case studies reinforce these lessons).
- Collaborate with patients and their families to develop specific action plans such as lifestyle medicine prescriptions (emphasized in weeks 2 and 3 with behavior change, goals, and accountability and addressed in each week thereafter).
- Help patients manage and sustain healthy lifestyle practices, including referrals as necessary (emphasized in week 1 with team approach to lifestyle medicine and discussed in weeks thereafter).

5. Office and community support (4 competencies)

- Have the ability to practice in interdisciplinary and community teams (emphasized in week 1 and discussed in weeks thereafter).
- Apply office systems and technologies to support of lifestyle medicine (mentioned but not emphasized as office systems vary, and this competency is the one that is the least applicable to the college population).
- Measure processes and outcomes (emphasized in week 1, and research on outcomes is addressed in each week’s material).
- 4. Use appropriate community referral resources to support implementation of healthy lifestyle (emphasized in week 1 and discussed throughout the course).

**Table 1:** Lifestyle Medicine Core Competencies met by the Lifestyle Medicine College Course syllabus.

Course Topic	Section Guiding Question and Topic Description	Competencies met by Course Topic
Week 1: Introduction and overview of lifestyle medicine	*Where am I now with healthy habits?* Discuss where students typically gain knowledge about healthy lifestyles and what are the preferred methods for gathering this important information. Have students reflect on their existing habits based on in-class assessment.	A1, A2, B1, B2, C1, C3, D4, E1, E3, and E4
Week 2: How to evoke behavior change for self and those you care for personally and professionally	*What motivates me and how do I respond when people try to help me?* Discussion centers around the coach approach. Have students compare and contrast their own experiences with health professionals using the expert and coach approaches.	A1, B1, B2, C1, C2, C3, D1, D2, D3, D4, EI, E3, and E4
Week 3: Goal setting, accountability, and tracking for lifestyle medicine	*What is a lifestyle goal that I want to work toward during this semester?* This section introduces the Health Behavior Change Project. Have students begin to consider how they will stay accountable and track progress.	A1, B1, B2, C1, C2, C3, D2, D3, D4, E1, E3, and E4
Week 4: Physical activity guidelines and prescription	*What is your level of physical activity?* Discussion centers on the development of an exercise prescription for ourselves and counseling others.	A1, B1, B2, C1, C2, C3, D1, D2, D3, D4, E1, E3, and E4
Week 5: Nutrition guidelines and prescription	*How can I eat a healthy diet while being a student this semester?* Introduces food tracking and food diaries. Discuss the Harvard Healthy Plate and compare/contrast with the recommendations of the US Department of Agriculture. Discuss cultural eating habits and other topics related to eating patterns (mindful eating, eating frequency, breakfast intake, etc).	A1, B1, B2, C1, C2, C3, D1, D2, D3, D4, E1, E3, and E4
Week 6: Sleep and its effect on health and well-being	*What is my sleep routine and how does it impact my body?* Discuss existing sleeping patterns in comparison with recommendations. Examine things that have an influence on sleep (emotions, nutrition, etc).	A1, B1, B2, C1, C2, C3, D1, D2, D3, D4, E1, E3, and E4
Week 7: Stress resilience	Sections: Guiding question— *How do I handle stress and what evidence-based techniques can I try to help me be more resilient?* Discussion of stress, eustress, the state of flow, and methods to combat stress reactions.	A1, B1, B2, C1, C2, C3, D1, D2, D3, D4, E1, E3, and E4
Week 8: Relaxation, mindfulness, and meditation	*How do I relax? What evidence-based techniques might I try in the future?* Review and introduce various relaxation techniques. Discuss techniques being used by students.	A1, B1, B2, C1, C2, C3, D1, D2, D3, D4, E1, E3, and E4
Week 9: The connection prescription	*What relationships do I cherish and why? How can I maintain social connections throughout my life?* Discuss benefits of connection and methods that can be used to increase and improve the level of connection.	A1, B1, B2, C1, C2, C3, D2, D3, D4, E1, E3, and E4
Week 10: Positive emotions: laughter, optimism, and gratitude	*What resonated most with you on the topic of positive emotions?* Practice expressing positive emotions in a group setting and create a plausible plan for fitting this into your daily routine.	A1, B1, B2, C1, C2, C3, D2, D3, D4, E1, E3, and E4
Week 11: Smoking, alcohol, and addiction	*How do you know when you are addicted?* Discuss how to help a person who smokes, drinks too much, or is addicted to drugs. Examine the controversial topic of “food addiction.”	A1, B1, B2, C1, C2, C3, D1, D2, D3, D4, E1, E3, and E4
Week 12: Self-care	*What do you do for self-care at the moment and what do you plan to do differently going forward?* Discuss the prioritization of self-care and methods to use. Use self-care assessment.	A1, B1, B2, C1, C2, C3, D2, D3, D4, E1, E3, and E4
Week 13: Education reform around lifestyle medicine and existing state of practice for lifestyle medicine	*If you could make a policy change that would encourage healthy lifestyle habits, what would it be and why?* Discuss and answer questions about the existing state of lifestyle medicine education and practice. Examine practical steps students can take locally.	A1, B1, B2, C1, C2, C3, D2, D3, D4, E1, E3, and E4

## Abbreviations

BMI: body mass index
SMART: specific, measurable, achievable, results-focused and time-bound

## References

[1] Kelly-Weeder S, Phillips K, Leonard K, Veroneau M (2014). Binge eating and weight loss behaviors of overweight and obese college students. J Am Assoc Nurse Pract.

[2] Greaney ML, Less FD, White AA, Dayton SF, Riebe D, Blissmer B, Shoff S, Walsh JR, Greene GW (2009). College students' barriers and enablers for healthful weight management: a qualitative study. J Nutr Educ Behav.

[3] Quick V, Byrd-Bredbenner C, White AA, Brown O, Colby S, Shoff S, Lohse B, Horacek T, Kidd T, Greene G (2014). Eat, sleep, work, play: associations of weight status and health-related behaviors among young adult college students. Am J Health Promot.

[4] Mahmoud AA, Warren KS (1975). Algorithms in the diagnosis and management of exotic diseases. ii. Giardiasis. J Infect Dis.

[5] Gropper SS, Simmons KP, Connell LJ, Ulrich PV (2012). Changes in body weight, composition, and shape: a 4-year study of college students. Appl Physiol Nutr Metab.

[6] Buscemi J, Yurasek AM, Dennhardt AA, Martens MP, Murphy JG (2011). A randomized trial of a brief intervention for obesity in college students. Clin Obes.

[7] Jin Y, Ding Z, Fei Y, Jin W, Liu H, Chen Z, Zheng S, Wang L, Wang Z, Zhang S, Yu Y (2014). Social relationships play a role in sleep status in Chinese undergraduate students. Psychiatry Res.

[8] Shi Z, Lien N, Kumar BN, Holmboe-Ottesen G (2005). Socio-demographic differences in food habits and preferences of school adolescents in Jiangsu Province, China. Eur J Clin Nutr.

[9] Lemma S, Gelaye B, Berhane Y, Worku A, Williams MA (2012). Sleep quality and its psychological correlates among university students in Ethiopia: a cross-sectional study. BMC Psychiatry.

[10] Yahia N, Achkar A, Abdallah A, Rizk S (2008). Eating habits and obesity among Lebanese university students. Nutr J.

[11] Al-Rethaiaa AS, Fahmy AA, Al-Shwaiyat NM (2010). Obesity and eating habits among college students in Saudi Arabia: a cross sectional study. Nutr J.

[12] Al-Nakeeb Y, Lyons M, Dodd L, Al-Nuaim A (2015). An investigation into the lifestyle, health habits and risk factors of young adults. Int J Environ Res Public Health.

[13] Goel M, Pal P, Agrawal A, Ashok C (2016). Relationship of body mass index and other life style factors with hypertension in adolescents. Ann Pediatr Cardiol.

[14] Djordjević-Nikić M, Dopsaj M, Vesković A (2013). Nutritional and physical activity behaviours and habits in adolescent population of Belgrade. Vojnosanit Pregl.

[15] Musaiger AO, Awadhalla MS, Al-Mannai M, AlSawad M, Asokan GV (2017). Dietary habits and sedentary behaviors among health science university students in Bahrain. Int J Adolesc Med Health.

[16] Musaiger AO, Awadhalla MS, Al-Mannai M, AlSawad M, Asokan GV (2017). Dietary habits and sedentary behaviors among health science university students in Bahrain. Int J Adolesc Med Health.

[17] Fabián C, Pagán I, Ríos JL, Betancourt J, Cruz SY, González AM, Palacios C, González MJ, Rivera-Soto WT (2013). Dietary patterns and their association with sociodemographic characteristics and perceived academic stress of college students in Puerto Rico. P R Health Sci J.

[18] Romaguera D, Tauler P, Bennasar M, Pericas J, Moreno C, Martinez S, Aguilo A (2011). Determinants and patterns of physical activity practice among Spanish university students. J Sports Sci.

[19] Chapman GE, Melton CL, Hammond G (1998). College and university students' breakfast consumption patterns: behaviours, beliefs, motivations and personal and environmental influences. Can J Diet Pract Res.

[20] Yahia N, Wang D, Rapley M, Dey R (2016). Assessment of weight status, dietary habits and beliefs, physical activity, and nutritional knowledge among university students. Perspect Public Health.

[21] Roberts KC, Danoff-Burg S (2010). Mindfulness and health behaviors: is paying attention good for you?. J Am Coll Health.

[22] Zivin K, Eisenberg D, Gollust SE, Golberstein E (2009). Persistence of mental health problems and needs in a college student population. J Affect Disord.

[23] White A, Hingson R (2013). The burden of alcohol use: excessive alcohol consumption and related consequences among college students. Alcohol Res.

[24] Borsari B, Hustad J, Mastroleo N, Tevyaw T, Barnett N, Kahler C, Short E, Monti PM (2012). Addressing alcohol use and problems in mandated college students: a randomized clinical trial using stepped care. J Consult Clin Psychol.

[25] Quinn PD, Fromme K (2011). Predictors and outcomes of variability in subjective alcohol intoxication among college students: an event-level analysis across 4 years. Alcohol Clin Exp Res.

[26] Caldeira KM, O'Grady KE, Garnier-Dykstra LM, Vincent KB, Pickworth WB, Arria AM (2012). Cigarette smoking among college students: longitudinal trajectories and health outcomes. Nicotine Tob Res.

[27] O'Grady KE, Arria AM, Fitzelle DM, Wish ED (2008). Heavy drinking and polydrug use among college students. J Drug Issues.

[28] Vella-Zarb RA, Elgar FJ (2009). The 'freshman 5': a meta-analysis of weight gain in the freshman year of college. J Am Coll Health.

[29] Cluskey M, Grobe D (2009). College weight gain and behavior transitions: male and female differences. J Am Diet Assoc.

[30] Crombie AP, Ilich JZ, Dutton GR, Panton LB, Abood DA (2009). The freshman weight gain phenomenon revisited. Nutr Rev.

[31] Lee S, Wuertz C, Rogers R, Chen Y (2013). Stress and sleep disturbances in female college students. Am J Health Behav.

[32] Vargas PA, Flores M, Robles E (2014). Sleep quality and body mass index in college students: the role of sleep disturbances. J Am Coll Health.

[33] Al-Rethaiaa AS, Fahmy AA, Al-Shwaiyat NM (2010). Obesity and eating habits among college students in Saudi Arabia: a cross sectional study. Nutr J.

[34] McComb S, Jones C, Smith A, Collins W, Pope B (2016). Designing incentives to change behaviors: examining college student intent toward healthy diets. West J Nurs Res.

[35] Kelly-Weeder S, Phillips K, Leonard K, Veroneau M (2014). Binge eating and weight loss behaviors of overweight and obese college students. J Am Assoc Nurse Pract.

[36] Kelly-Weeder S, Phillips K, Leonard K, Veroneau M (2014). Binge eating and weight loss behaviors of overweight and obese college students. J Am Assoc Nurse Pract.

[37] Lowry R, Galuska DA, Fulton JE, Wechsler H, Kann L, Collins J (2000). Physical activity, food choice, and weight management goals and practices among US college students. Am J Prev Med.

[38] Moreno L, Gottrand F, Huybrechts I, Ruiz J, González-Gross M, DeHenauw S, HELENA Study Group (2014). Nutrition and lifestyle in European adolescents: the HELENA (healthy lifestyle in Europe by nutrition in adolescence) study. Adv Nutr.

[39] Regehr C, Glancy D, Pitts A (2013). Interventions to reduce stress in university students: a review and meta-analysis. J Affect Disord.

[40] Kelly NR, Mazzeo SE, Bean MK (2013). Systematic review of dietary interventions with college students: directions for future research and practice. J Nutr Educ Behav.

[41] Hong MY, Shepanski TL, Gaylis JB (2016). Majoring in nutrition influences BMI of female college students. J Nutr Sci.

[42] Musaiger AO, Awadhalla MS, Al-Mannai M, AlSawad M, Asokan GV (2017). Dietary habits and sedentary behaviors among health science university students in Bahrain. Int J Adolesc Med Health.

[43] Vargas EJ, Zelis R (2014). Integrating nutrition education into the cardiovascular curriculum changes eating habits of second-year medical students. J Clin Lipidol.

[44] American College of Lifestyle Medicine (2016). Lifestylemedicine.

[45] American College of Lifestyle Medicine (2016). Lifestylemedicine.

[46] Poddar KH, Hosig KW, Anderson ES, Nickols-Richardson SM, Duncan SE (2010). Web-based nutrition education intervention improves self-efficacy and self-regulation related to increased dairy intake in college students. J Am Diet Assoc.

[47] Office of Disease Prevention and Health Promotion (2016). Health.gov.

[48] Mokdad AH, Marks JS, Stroup DF, Gerberding JL (2004). Actual causes of death in the United States, 2000. JAMA.

[49] Song M, Giovannucci E (2016). Preventable incidence and mortality of carcinoma associated with lifestyle factors among white adults in the United States. JAMA Oncol.

[50] Stutts JC, Wilkins JW, Scott Osberg J, Vaughn BV (2003). Driver risk factors for sleep-related crashes. Accid Anal Prev.

[51] Wheaton AG, Olsen EO, Miller G, Croft JB (2016). Sleep duration and injury-related risk behaviors among high school students? United States, 2007-2013. MMWR Morb Mortal Wkly Rep.

[52] Pirraglia PA, Hampton JM, Rosen AB, Witt WP (2011). Psychological distress and trends in healthcare expenditures and outpatient healthcare. Am J Manag Care.

[53] Cohen S, Janicki-Deverts D, Miller GE (2007). Psychological stress and disease. JAMA.

[54] Csikzentmihalyi M (2014). Flow and the Foundations of Positive Psychology: The Collected Works of Mihaly Csikszentmihalyi.

[55] Rubak S, Sandbaek A, Lauritzen T, Christensen B (2005). Motivational interviewing: a systematic review and meta-analysis. Br J Gen Pract.

[56] Trajkovski S, Schmied V, Vickers M, Jackson D (2013). Using appreciative inquiry to transform health care. Contemp Nurse.

[57] Rubak S, Sandbaek A, Lauritzen T, Christensen B (2005). Motivational interviewing: a systematic review and meta-analysis. Br J Gen Pract.

[58] Puhl R, Heuer CA (2010). Obesity stigma: important considerations for public health. Am J Public Health.

[59] Lianov L, Johnson M (2010). Physician competencies for prescribing lifestyle medicine. JAMA.

[60] Abramson S, Stein J, Schaufele M, Frates E, Rogan S (2000). Personal exercise habits and counseling practices of primary care physicians: a national survey. Clin J Sport Med.

[61] Frank E, Tong E, Lobelo F, Carrera J, Duperly J (2008). Physical activity levels and counseling practices of U.S. medical students. Med Sci Sports Exerc.

